# Time Preferences Predict Mortality among HIV-Infected Adults Receiving Antiretroviral Therapy in Kenya

**DOI:** 10.1371/journal.pone.0145245

**Published:** 2015-12-18

**Authors:** Harsha Thirumurthy, Kami Hayashi, Sebastian Linnemayr, Rachel C. Vreeman, Irwin P. Levin, David R. Bangsberg, Noel T. Brewer

**Affiliations:** 1 Department of Health Policy and Management, Gillings School of Global Public Health, University of North Carolina at Chapel Hill, Chapel Hill, NC, United States of America; 2 Carolina Population Center, University of North Carolina at Chapel Hill, Chapel Hill, NC, United States of America; 3 Department of Health Behavior, Gillings School of Global Public Health, University of North Carolina at Chapel Hill, Chapel Hill, NC, United States of America; 4 RAND Corporation, Santa Monica, CA, United States of America; 5 Department of Pediatrics, Indiana University School of Medicine, Indianapolis, IN, United States of America; 6 Academic Model Providing Access to Healthcare, Eldoret, Kenya; 7 Department of Psychology, University of Iowa, Iowa City, IA, United States of America; 8 Center for Global Health, Massachusetts General Hospital, Harvard Medical School, Boston, MA, United States of America; CEA, FRANCE

## Abstract

**Background:**

Identifying characteristics of HIV-infected adults likely to have poor treatment outcomes can be useful for targeting interventions efficiently. Research in economics and psychology suggests that individuals’ intertemporal time preferences, which indicate the extent to which they trade-off immediate vs. future cost and benefits, can influence various health behaviors. While there is empirical support for the association between time preferences and various non-HIV health behaviors and outcomes, the extent to which time preferences predict outcomes of those receiving antiretroviral therapy (ART) has not been examined previously.

**Methods:**

HIV-infected adults initiating ART were enrolled at a health facility in Kenya. Participants’ time preferences were measured at enrollment and used to classify them as having either a low or high discount rate for future benefits. At 48 weeks, we assessed mortality and ART adherence, as measured by Medication Event Monitoring System (MEMS). Logistic regression models adjusting for socio-economic characteristics and risk factors were used to determine the association between time preferences and mortality as well as MEMS adherence ≥90%.

**Results:**

Overall, 44% (96/220) of participants were classified as having high discount rates. Participants with high discount rates had significantly higher 48-week mortality than participants with low discount rates (9.3% vs. 3.1%; adjusted odds ratio 3.84; 95% CI 1.03, 14.50). MEMS adherence ≥90% was similar for participants with high vs. low discount rates (42.3% vs. 49.6%, AOR 0.70; 95% CI 0.40, 1.25).

**Conclusion:**

High discount rates were associated with significantly higher risk of mortality among HIV-infected patients initiating ART. Greater use of time preference measures may improve identification of patients at risk of poor clinical outcomes. More research is needed to further identify mechanisms of action and also to build upon and test the generalizability of this finding.

## Introduction

Despite the significant health improvements achieved as a result of the successful scale-up of antiretroviral therapy (ART) in sub-Saharan Africa, mortality rates have been high among HIV-infected adults receiving ART, particularly in the first year of treatment [1–3]. While early mortality has partly been the result of ART initiation at low CD4+ T-cell counts [1, 2], studies have also identified individual and health system factors affecting mortality [2] as well as adherence and retention in care [4–6]. Research in economics and psychology highlights other potentially important individual-level predictors of health behavior, and there has recently been heightened interest in utilizing insights from these disciplines to interpret and influence behaviors related to HIV prevention and treatment. An important in models of decision-making has been individuals’ intertemporal time preferences, which indicate how they weigh immediate vs. future costs and benefits. By describing the extent to which an individual discounts delayed outcomes, time preferences have been hypothesized to be an important predictor of health behaviors that have immediate costs (or benefits) but delayed rewards (or costs) [7]. Across many studies of non-HIV related health behaviors, individuals’ time preferences have predicted health behaviors with immediate costs and delayed benefits, such as smoking [8–10], alcohol use [11–13], obesity [14], hypertension management [15], adherence to asthma-control medication [16], and sexual risk behavior [17]. However, some studies have found no relationship between time preferences and health behaviors such as vaccination [18, 19]. However, the extent to which time preferences predict outcomes of those receiving ART has not been examined previously.

Adherence to ART and retention in care require incurring present and immediate costs (e.g., time and travels costs to seek care, adverse side effects, and inconvenience of taking daily medication) in order to realize future benefits (e.g., living longer, being able to work, and being less likely to infect sexual partners with HIV) [4, 6]. Thus, it is plausible that outcomes of individuals receiving ART would be worse for those who display higher discounting of delayed outcomes. However, time preferences are rarely measured in patient encounter forms or studies that focus on outcomes of those on ART. Examining the association between patients’ time preferences and outcomes such as adherence and mortality can suggest novel ways of identifying patients needing outreach and support following ART initiation, thereby leading to improvements in clinical outcomes and program cost-effectiveness.

This study examined whether the time preferences of HIV-infected adults receiving ART are associated with mortality and ART adherence over a 48-week period.

## Methods

### Participants

Data were collected within a randomized controlled trial (RCT) that tested whether mobile phone text messages improved ART adherence (ClinicalTrials.gov identifier NCT01058694) [20]. The study site was a government-run health facility in Kenya’s Nyanza region where the USAID-Academic Model Providing Access to Healthcare (AMPATH) provided free comprehensive care to HIV-infected patients. Patients who were aged >18 years, HIV-infected, and were either initiating ART at the time of enrollment or had initiated ART within the past 3 months were eligible to participate in the RCT. There were no other eligibility criteria for the RCT. Patients who met eligibility criteria and provided informed consent were provided with a mobile phone and randomized to receive different types of daily or weekly text messages that encouraged ART adherence or to a control group that received no text messages. This study focused on the control group in the RCT, which did not receive the text message interventions. Study participants were enrolled between June 2007 and August 2008.

### Procedures

The RCT procedures have been described in greater detail previously [20]. At the time of enrollment, participants completed a survey that assessed demographic characteristics and time preferences (described below). Study staff at the clinic’s pharmacy transferred one of their three antiretroviral medications to a pill bottle equipped with a Medication Event Monitoring System (MEMS) cap (Aardex Group, Switzerland) that electronically recorded the date and time of each opening. Participants were expected to return to the clinic each month per standard clinic protocol, and study staff downloaded MEMS data at each return visit. Participants were followed for 48 weeks after enrollment. The Institutional Research Ethics Committee of the Moi University School of Medicine and the Institutional Review Boards of Georgetown University and the University of North Carolina at Chapel Hill approved the study. The study used informed consent forms and procedures that were approved by the ethics committees. All participants provided written informed consent to participate in the study. Signed consent forms were retained by the research assistants who obtained informed consent and then stored in secure facilities. Participants were also provided with a copy of the signed consent forms.

### Measures

#### Outcomes

The main outcome for this secondary analysis was mortality at 48 weeks after enrollment. Information on mortality was obtained by study staff based on a review of clinic records. ART adherence was calculated as the number of MEMS-recorded bottle openings divided by the number of prescribed doses until MEMS data were unavailable due to mortality or loss to follow-up. The number of daily bottle openings was limited to two in order to avoid overstating adherence due to extraneous bottle openings. We then defined a binary indicator of adherence ≥90% during the study period.

#### Time preferences

Participants were asked to choose between *hypothetical* monetary payments made to them either on the day of the interview or in one year, a standard method of eliciting intertemporal time preferences. Participants were asked to suppose they had just won a prize that will give them a series of payments over time and asked whether they would choose to receive 550 Kenya Shillings (about US$7.00) immediately or 1,000 Kenya Shillings (US$12.50) in one year. Participants indicating they would choose the immediate reward were classified as having a high discount rate (i.e., displaying more impatience and less future connectedness), while those indicating they would choose the delayed reward were classified as having a low discount rate (i.e. less impatience and more future connectedness).

### Statistical analyses

We used logistic regression analyses to examine the association between participants’ time preferences and mortality as well as adherence. In the unadjusted model, the two outcome variables were regressed on the binary indicator of whether a participant had a high discount rate. In the adjusted model, participants’ age, sex, education, marital status, household size, travel time to clinic, wealth (measured by indicator of house with iron roof), alcohol use, and an indicator of whether participants reported feeling tired or lacking energy in the past week (a measure of health status at the time of ART initiation) were also included as covariates. All analyses were conducted with STATA version 13.1.

## Results

Among 231 participants in the control group of the RCT, 11 were excluded from the analyses due to missing data on time preferences and other variables included in the model. For the remaining 220 participants, over two thirds were female and the average age was 36.1 years (Table 1). The average travel time to the ART clinic was 1.5 hours. About half the participants reported feeling tired or lacking energy in the past week. Nearly one-half of participants had high discount rates (43.6%, 96/220).

**Table 1:** Participant characteristics and outcomes.

		Time preferences		
	All participants	Low discount rate	High discount rate	P-value
N	220	124	96	
**Participant characteristics**				
Age, in years (mean)	36.1	36.3	35.7	0.65
Female	68.6%	65.1%	73.2%	0.20
Married or cohabitating	45.6%	47.3%	43.3%	0.55
Widowed	38.1%	36.4%	40.2%	0.57
Household size	6.5	6.4	6.6	0.66
Completed primary school	56.6%	52.7%	61.9%	0.17
House has iron roof	84.5%	82.2%	87.6%	0.26
Travel time to clinic, in hours (mean)	1.5	1.5	1.4	0.78
Tired or lacking energy in past week	51.7%	47.6%	57.3%	0.15
Sometimes drinks alcohol	8.0%	8.5%	7.2%	0.72
**Outcomes**				
MEMS adherence ≥90% over 48 weeks	45.9%	49.6%	42.3%	0.28
Deceased at 48 weeks	5.9%	3.1%	9.3%	0.05

Notes: P-values are from chi-squared tests comparing characteristics of participants with low and high discount rates (*t*-tests for continuous variables). Participants were classified as having a low or high discount rate discount rate based on their choice between hypothetical monetary payments made either on the day of the interview or in one year, a standard method of eliciting intertemporal time preferences. Abbreviations: MEMS, medication event monitoring system.

At 48 weeks, 5.9% (13/220) participants were deceased. There was a sizable difference in mortality between participants with low and high discount rates (3.1% and 9.3%, respectively). In regression analysis that included adjustment for covariates (Table 2), participants with high discount rates were significantly more likely to be deceased at 48 weeks than those with low discount rates (adjusted odds ratio (AOR) 3.84; 95% CI 1.03, 14.50).

**Table 2:** Association between time preferences, mortality, and MEMS adherence.

	Deceased at 48 weeks		MEMS Adherence ≥90%	
	Odds ratio (95% CI)		Odds ratio (95% CI)	
High discount rate	3.19 (1.02–10.70)	3.84 (1.03–14.50)	0.74 (0.44–1.26)	0.70 (0.40–1.25)
Age				
18–24		-		Reference
25–34		0.90 (0.14–5.86)		1.48 (0.55–4.00)
35–44		1.28 (0.17–9.61)		2.16 (0.76–6.13)
45 and above		0.92 (0.09–9.37)		1.79 (0.58–5.51)
Female		0.21 (0.04–1.06)		1.64 (0.75–3.59)
Married or cohabitating		0.21 (0.04–1.28)		1.54 (0.61–3.88)
Widowed		0.19 (0.03–1.41)		1.17 (0.46–2.98)
Household size		1.07 (0.84–1.37)		1.09 (0.96–1.23)
Completed primary school		0.97 (0.25–3.74)		0.72 (0.39–1.33)
House has iron roof		0.94 (0.16–5.33)		1.75 (0.75–4.06)
Travel time to clinic (hours)		1.14 (0.69–1.86)		0.89 (0.68–1.16)
Tired or lacking energy in past week		2.42 (0.61–9.59)		1.18 (0.64–2.13)
Sometimes drinks alcohol		0.85 (0.08–9.37)		0.29 (0.07–1.12)

Notes: Results from logistic regression models. Outcome variable in columns (1) and (2) is a binary indicator of mortality of 48 weeks and in columns (3) and (4) is a binary indicator of 48-week MEMS adherence ≥90%. The binary variable “high discount rate” indicates whether the participant chose to receive hypothetical monetary payments on the day of the interview rather than in one year, a standard method of eliciting intertemporal time preferences. Abbreviations: CI, confidence interval; MEMS, medication event monitoring system.

Nearly half (45.9%, 101/220) the participants achieved MEMS adherence ≥90% during the 48-week study period. MEMS adherence ≥90% was achieved by 42.3% of participants with a high discount rate and 49.6% of participants with a low discount rate. However, participants’ discount rates were not associated with MEMS adherence in the regression analysis (AOR 0.70; 95% CI 0.40–1.25).

Participants with low discount rates did appear to have better health status at the time of ART initiation than those with high discount rates (47.3% reporting feeling tired or lacking energy compared to 57.3%). This baseline measure of health status and other covariates included in the regression model had the expected direction of associations with mortality and adherence, but the associations were generally not statistically significant.

## Discussion

A simple measure of HIV-infected adults’ time preferences obtained near the time of ART initiation was used to classify participants as having either low or high discount rates. Participants with high discount rates had significantly higher 48-week mortality, an association that has not been documented previously in the literature on HIV treatment outcomes. These patients also had lower ART adherence but the association was not statistically significant. Despite the small sample size in this study, the findings suggest that measuring patients’ time preferences at the time of ART initiation may be useful for identifying those who are particularly in need of clinical support and monitoring. Further measurement and analysis of time preferences among larger populations of patients receiving ART, with the inclusion of additional measures of clinical outcomes during various stages of treatment, may yield valuable insights on how to target adherence interventions and which interventions to choose.

The finding that 48-week mortality, which was similar to rates reported in other studies [21], was significantly higher for those with high discount rates is a striking result that reveals the potential value of measuring time preferences at the time of ART initiation. This result was robust to controls for other risk factors that may have been correlated with time preferences, such as wealth and health status at the time of ART initiation.

Medication adherence is an *a priori* reason why patients’ time preferences may predict clinical outcomes after ART initiation, since adherence involves incurring short-term costs in order to achieve long-term health benefits. Interventions that can promote ART adherence vary considerably in cost and complexity [22], resulting in a need for care programs to target interventions selectively. Since time preference measures can be obtained from patients at the time of ART initiation and are feasible to implement even among individuals with low education, they may enable programs to allocate more resource-intensive interventions to patients with high discount rates, much in the way that patients’ reports of substance abuse or depression are sometimes used to identify support needs. For patients with high discount rates in particular, the rationale for using financial incentives as an intervention to improve adherence may also be stronger. Given the growing interest in behavioral economics and in using incentives to promote adherence and retention in care [23], this study suggests ways to identify patients who may benefit the most from such interventions.

Paired with the results for mortality, the lack of a significant association between time preferences and adherence is intriguing. Our result that patients with high discount rates have lower adherence is consistent with the finding of higher mortality for these patients, but the lack of a significant association may be due to limited statistical power. However, the result may be consistent with some previous research that time preferences are associated with “hot” or impulsive behaviors such as drug use and gambling but not with “cold” behaviors such as medication adherence or vaccination uptake [19]. Thus, other mediators apart from adherence that were not studied here may better explain why time preferences predict mortality. The mechanisms explaining the association of time preferences and mortality require further investigation.

Key strengths of this study include a clinical population in a high prevalence setting, a long follow-up period, objective adherence measures, and innovative use of a simple time preference measure to predict outcomes. Importantly, the time preference measure used in this study resembles measures used in other studies conducted in resource-limited settings and was easy to implement even among patients with low literacy or numeracy. The results were also robust to controlling for individuals’ socio-economic status, which can often be associated with time preference measures. Limitations include the relatively small sample size of patients, which may have limited statistical power and generalizability. Second, the results may be sensitive to the particular time preference measures used. While there is no gold standard for measuring time preferences [24], more detailed measures could classify patients into several categories of discount rates. The feasibility of using more burdensome measurement strategies in high-volume clinics may be limited, however. One concern about asking participants to make choices between hypothetical monetary payments in the present vs. the future is that in a low-income population, the choices may reflect current economic circumstances rather than discount rates. While we cannot rule out this possibility and time preference measures can be sensitive to economic circumstances, the robustness of the main result to inclusion of controls for wealth suggests that this is not a major source of bias. This study also did not measure whether participants displayed time-inconsistent (or present-biased) preferences, which have been hypothesized in the behavioral economics literature as an additional barrier to behaviors involving immediate costs and delayed benefits [25, 26]. Implementing additional measures of time preferences and testing their association with additional clinical outcomes could offer valuable new insights. Finally, bias stemming from residual confounding remains a possibility despite our attempts to control for various individual characteristics. Although we did not control for baseline CD4 counts, which were unavailable in this study, we did include a self-reported measure of health status at the time of ART initiation. It is noteworthy, therefore, that time preferences remained significantly associated with mortality even after controlling for this health measure.

Our findings show that even simple measures of time preferences can predict mortality among HIV-infected adults receiving ART. Further analysis of ways in which patients’ time preferences predict clinical outcomes may provide valuable guidance for programs that provide care to growing numbers of patients and seek to increase the efficiency with which they provide treatment support. Implementing simple measures of time preferences among larger cohorts of adults receiving ART and assessing their association with key treatment outcomes is an important next step for research in this area. Time preference measures may also be useful for predicting HIV risk behaviors.

## References

[1] Boulle A, Schomaker M, May MT, Hogg RS, Shepherd BE, Monge S, et al Mortality in Patients with HIV-1 Infection Starting Antiretroviral Therapy in South Africa, Europe, or North America: A Collaborative Analysis of Prospective Studies. PLoS Med 2014,11:e1001718 10.1371/journal.pmed.1001718 25203931 PMC4159124

[2] May M, Boulle A, Phiri S, Messou E, Myer L, Wood R, et al Prognosis of patients with HIV-1 infection starting antiretroviral therapy in sub-Saharan Africa: a collaborative analysis of scale-up programmes. The Lancet 2010,376:449–457. 10.1016/S0140-6736(10)60666-6 PMC3138328 20638120

[3] Braitstein P, Brinkhof MW, Dabis F, Schechter M, Boulle A, The Antiretroviral Therapy in Lower Income Countries (ART-LINC) Collaboration. Mortality of HIV-1-infected patients in the first year of antiretroviral therapy: comparison between low-income and high-income countries. The Lancet 2006,367:817–824. 10.1016/S0140-6736(06)68337-2 16530575

[4] Mills EJ, Nachega JB, Bangsberg DR, Singh S, Rachlis B, Wu P, et al Adherence to HAART: a systematic review of developed and developing nation patient-reported barriers and facilitators. PLoS Med 2006,3:e438 17121449 10.1371/journal.pmed.0030438 PMC1637123

[5] Siedner MJ, Lankowski A, Tsai AC, Muzoora C, Martin JN, Hunt PW, et al GPS-measured distance to clinic, but not self-reported transportation factors, are associated with missed HIV clinic visits in rural Uganda. AIDS 2013,27:1503–1508. 10.1097/QAD.0b013e32835fd873 23435294 PMC3745818

[6] Ware NC, Idoko J, Kaaya S, Biraro IA, Wyatt MA, Agbaji O, et al Explaining adherence success in sub-Saharan Africa: an ethnographic study. PLoS Med 2009,6:e11 10.1371/journal.pmed.1000011 19175285 PMC2631046

[7] Frederick S, Loewenstein G, O'Donoghue T. Time discounting and time preference: a critical review. Journal of Economic Literature 2002,40:351–401.

[8] Baker F, Johnson MW, Bickel WK. Delay discounting in current and never-before cigarette smokers: similarities and differences across commodity, sign, and magnitude. J Abnorm Psychol 2003,112:382–392. 12943017 10.1037/0021-843x.112.3.382

[9] Bickel WK, Yi R, Kowal BP, Gatchalian KM. Cigarette smokers discount past and future rewards symmetrically and more than controls: is discounting a measure of impulsivity? Drug Alcohol Depend 2008,96:256–262. 10.1016/j.drugalcdep.2008.03.009 18468814 PMC2701143

[10] Odum AL, Madden GJ, Bickel WK. Discounting of delayed health gains and losses by current, never- and ex-smokers of cigarettes. Nicotine Tob Res 2002,4:295–303. 12215238 10.1080/14622200210141257

[11] Petry NM. Delay discounting of money and alcohol in actively using alcoholics, currently abstinent alcoholics, and controls. Psychopharmacology (Berl) 2001,154:243–250. 11351931 10.1007/s002130000638

[12] MacKillop J, Amlung MT, Few LR, Ray LA, Sweet LH, Munafo MR. Delayed reward discounting and addictive behavior: a meta-analysis. Psychopharmacology (Berl) 2011,216:305–321. 21373791 10.1007/s00213-011-2229-0 PMC3201846

[13] MacKillop J, Miranda R Jr., Monti PM, Ray LA, Murphy JG, Rohsenow DJ, et al Alcohol demand, delayed reward discounting, and craving in relation to drinking and alcohol use disorders. J Abnorm Psychol 2010,119:106–114. 10.1037/a0017513 20141247 PMC2862264

[14] Smith PK, Bogin B, Bishai D. Are time preference and body mass index associated?: Evidence from the National Longitudinal Survey of Youth. *Economics &* Human Biology 2005,3:259–270. 10.1016/j.ehb.2005.05.001 15964787

[15] Axon RN, Bradford WD, Egan BM. The role of individual time preferences in health behaviors among hypertensive adults: a pilot study. J Am Soc Hypertens 2009,3:35–41. 10.1016/j.jash.2008.08.005 20409943

[16] Brandt S, Dickinson B. Time and Risk Preferences and the Use of Asthma Controller Medication. Pediatrics 2013,131:e1204–e1210. 10.1542/peds.2011-2982 23478866

[17] MacKillop J, Celio MA, Mastroleo NR, Kahler CW, Operario D, Colby SM, et al Behavioral economic decision making and alcohol-related sexual risk behavior. AIDS and Behavior 2015,19:450–458. 10.1007/s10461-014-0909-6 25267115 PMC4359027

[18] Chapman GB, Brewer NT, Coups EJ, Brownlee S, Leventhal H, Leventhal EA. Value for the future and preventive health behavior. *Journal of experimental psychology* . Applied 2001,7:235–250. 11676102

[19] Chapman GB. Short-term cost for long-term benefit: time preference and cancer control. Health psychology: official journal of the Division of Health Psychology, American Psychological Association 2005,24:S41–48. 10.1037/0278-6133.24.4.S41 16045418

[20] Pop-Eleches C, Thirumurthy H, Habyarimana JP, Zivin JG, Goldstein MP, de Walque D, et al Mobile phone technologies improve adherence to antiretroviral treatment in a resource-limited setting: a randomized controlled trial of text message reminders. AIDS 2011,25:825–834. 10.1097/QAD.0b013e32834380c1 21252632 PMC3718389

[21] Wools-Kaloustian K, Kimaiyo S, Diero L, Siika A, Sidle J, Yiannoutsos CT, et al Viability and effectiveness of large-scale HIV treatment initiatives in sub-Saharan Africa: experience from western Kenya. AIDS 2006,20:41–48. 16327318 10.1097/01.aids.0000196177.65551.ea

[22] Barnighausen T, Chaiyachati K, Chimbindi N, Peoples A, Haberer J, Newell ML. Interventions to increase antiretroviral adherence in sub-Saharan Africa: a systematic review of evaluation studies. Lancet Infect Dis 2011,11:942–951. 10.1016/S1473-3099(11)70181-5 22030332 PMC4250825

[23] Galarraga O, Genberg BL, Martin RA, Barton Laws M, Wilson IB. Conditional economic incentives to improve HIV treatment adherence: literature review and theoretical considerations. AIDS Behav 2013,17:2283–2292. 10.1007/s10461-013-0415-2 23370833 PMC3688660

[24] Gyrd-Hansen D. Comparing the results of applying different methods of eliciting time preferences for health. European Journal of Health Economics 2002,3:10–16. 15609113 10.1007/s10198-002-0098-5

[25] Loewenstein G, Brennan T, Volpp KG. Asymmetric paternalism to improve health behaviors. JAMA 2007,298:2415–2417. 18042920 10.1001/jama.298.20.2415

[26] O’Donoghue T, Rabin M. Doing it now or later. American Economic Review 1999,89:103–124.

